# Supplementary material for: All-optical temporal integration mediated by subwavelength heat antennas
Source: Nat Commun. 2025 Dec 23;17:989. doi: 10.1038/s41467-025-67726-0 (PMC12847805; doi:10.1038/s41467-025-67726-0)
Supplement: Supplementary file 1 — Supplementary Information [file 41467_2025_67726_MOESM1_ESM.pdf]

Supplementary Information for

All-optical temporal integration mediated by subwavelength heat antennas

Yi Zhang[1]†, Nikolaos Farmakidis[1]†, Ioannis Roumpos[2]†, Miltiadis Moralis-Pegios[3], Apostolos Tsakyridis[3], June Sang Lee[1], Bowei Dong[1], Yuhan He[1], Samarth Aggarwal[1], Nikos Pleros[3]* and Harish Bhaskaran[1]*

Corresponding authors: harish.bhaskaran@materials.ox.ac.uk, npleros@csd.auth.gr

Supplementary Note 1. Device architecture and optical path design

Supplementary Fig. 1 presents the optical microscope and scanning electron microscope (SEM) images of the Photonic-Heater-in-Lightpath (PHIL) ring resonator, together with the schematic of its excitation and output configuration. The device is based on a silicon-on-insulator (SOI) microring resonator that incorporates a pair of absorbers deposited directly on the waveguide. These nanoheaters are aligned with the standing-wave antinodes formed by the interference of counter-propagating modes, allowing precise and localized optical absorption.

Light from the input port is divided by a 1:2 multimode interference (MMI) splitter, which generates two counter-propagating optical paths that simultaneously excite the microring. The resulting clockwise and counter-clockwise modes establish a standing-wave field distribution inside the ring cavity, which is crucial for enabling position-dependent absorption and phase modulation. By engineering the heater locations at the antinodes, the optical power is efficiently converted into localized heat, thereby inducing a controllable thermo-optic phase shift without relying on external electrical drivers.

The out-coupled signals from the ring are symmetrically distributed between two output waveguides, corresponding to the drop-port geometry of the interferometric configuration.

Supplementary Note 2. Simulation of spectral-selective absorption

Spectral-selective absorption is illustrated using finite difference time domain simulations (FDTD, Lumerical solutions) in Supplementary Fig. 2a. Here, the spatial field distribution in a silicon rib waveguide with absorptive titanium antennas on top shows periodic nodes and antinodes when coherent light is coupled in counterpropagating directions. For two antiphase wavelengths λ_1 and λ_2 the field at the antenna shows a maximum and a minimum respectively (Supplementary Fig. 2b, c). This wavelength selectivity is reflected in the absorption profiles (Supplementary Fig.

2d), where we calculate the total power absorbed by the antenna by integrating over the height of the structure. Here the total absorption experienced by λ_1 is 17 times higher than that of λ_2, where the structure is nearly transparent; any loss at λ_2 results from the finite size of the antenna.

As shown in Supplementary Fig. 2e, f, after sending a 1-mW pulse with 500 ns width, the temperature between two antennas can be raised to 325.5 K with input λ_1, while its counterpart with input λ_2 remains around room temperature (301.1 K). This demonstrates the spatial non-locality of the thermo-optic effect induced by power absorption in nano-antennas. It also indicates low optical loss for wavelengths that do not overlap with the nano-antennas, thereby confirming the non-reciprocal behaviour predicted by our design.

Supplementary Note 3. Evaluation of device uniformity

To evaluate device-to-device uniformity of the OTO effect, ten PHIL units with identical design parameters were fabricated and tested. The OTO response was characterized by quantifying the resonance spectrum shift under excitation from a calibrated pump laser, with all measurements performed under identical conditions (Supplementary Fig. 3). Across the measured devices, a highly linear dependence of phase shift on pump power was consistently observed, confirming reproducible and uniform OTO behaviour. The extracted phase tuning efficiencies exhibit a mean slope of 0.040 π/mW with minimal standard deviation of 0.002π (5.3% variation).

Supplementary Note 4. Pump at the non-absorptive wavelength

We changed the control line to the even-mode-resonant wavelength with similar transmission and repeated the process above to collect spectrums around the odd-mode-resonant peak as shown in the Supplementary Fig. 4a. Pumping at the probe signal (even-mode-resonant wavelength) has a weaker effect on shifting the spectrum at 0.0087 π/mW (Supplementary Fig. 4b) compared with 0.04 π/mW while pumping at the control signal (odd-mode-resonant wavelength), which reconfirms the low absorption at the probe signal and non-reciprocal operation proposed theoretically above.

Supplementary Note 5. Bit resolution analysis

To quantify the analogue precision of the PHIL device under multi wavelength-division multiplexing (WDM) channels, we evaluate the effective bit resolution (BR) based on the statistical variability of the measured optical response. The dynamic bit resolution is defined as:

$$\text{Bit Resolution(BR)} = \log_2\left(\frac{1}{\sigma}\right)$$

where σ is the standard deviation of the normalized output error with respect to the ideal additive or integrated response. This metric is equivalent to the effective number of bits (ENOB) in analog systems and serves as a measure of precision for both static and dynamic photonic computation.

Precision with a Single WDM Channel:

We evaluated the bit resolution by quantifying the variability of the integrated output under randomized control-signal (CS) pulse trains, and the peak resolution was achieved near the optimal absorption powers (~1.5–1.7 mW), with a mean ENOB of 4.9 bits across the tested power range. This precision reflects the combined influence of photodetector noise, thermal fluctuations, and absorption-induced nonlinearity, indicating stable and reproducible time-domain integration in a single-channel configuration.

Precision under Two WDM Channels (Static Modulation):

We conducted controlled two-channel WDM summation experiments, where two independent CS wavelengths were simultaneously modulated. The measured output agrees closely with the expected additive response, and the corresponding error histogram yields a standard deviation of $\sigma = 0.0181$ with near-zero mean bias. This corresponds to an effective precision of approximately 5–6 bits, demonstrating the excellent linearity and additive behaviour of the PHIL device under static multi-wavelength excitation.

Precision under Three WDM Channels (Static Modulation):

To further evaluate how the effective resolution scales with the number of simultaneous wavelength channels, we extended the WDM summation experiment to a three-wavelength configuration (Supplementary Fig. 7).

The resulting error histograms (Supplementary Fig. 7c) yield standard deviations of $\sigma \approx 0.04$, depending on the fixed input level. This corresponds to an effective resolution of ≈ 4.5 bits, compared with the 5–6 bits obtained in the two-channel case. The moderate reduction in precision primarily arises from increased thermal loading and thermo-optic crosstalk among multiple absorptive channels interacting within the shared microring cavity.

Importantly, the overall device response remains highly linear, and the bit precision continues to fall within a usable analogue range for neuromorphic photonic computation. We anticipate that improved thermal isolation, optimized nanoheater geometries, and balanced input-power normalization will further mitigate inter-channel coupling in future device generations, enabling higher-bit operation under large-scale WDM operation.

Supplementary Note 6. Response characterization & leaky integrator

The modulation results on the probe signal transmission with different control signal pulse width are shown in Supplementary Fig. 9a, which has a response similar to the output of a leaky integrator due to the slow heat dissipation process.

In a leaky integrator, if we define *y(t)* as the output of this system at any time *t*,

$$y(t) \propto e^{-\frac{t}{\tau}}$$

The impulse response of a leaky integrator decays exponentially, in which the leak time constant (τ) defines how quickly the integrator "forgets" past inputs due to the leakage and determines the integration window of the system. τ is typically defined as the time it takes to decay to approximately 1/e of its initial value when the input is removed. In our case, therefore, we estimate the time constant with device response to a 200-ns step input in Supplementary Fig. 9a, when the device response reaches the steady state. Supplementary Fig. 9b shows a zoomed response characterization at 200 ns control pulse width and its extracted time constant (τ) is 130 ns, indicating a theoretical integration window where data can be added up before being 'forgotten', i.e. 'leaked'.

Supplementary Note 7. MNIST task implementation

The experimentally measured PHIL transfer curve was normalized to the range [–0.2, 1.0] and implemented as a piecewise-linear differentiable activation in PyTorch (Supplementary Fig. 11a). The activation was integrated into a fully connected network (784–256–128–10) for the MNIST handwritten-digit classification task, replacing the standard ReLU function (Supplementary Fig. 11b). Training used the Adam optimizer (lr = 3×10^{-4}, 20 epochs, batch = 64) with adaptive rescaling to match the PHIL input range. The dataset was pre-normalized to [–0.2, 1.0].

The PHIL-activated network achieved a test accuracy of 97.2 %, compared with 97.8 % for ReLU (Supplementary Fig. 11c–e), showing nearly identical convergence and uniform class accuracy. These results confirm that the experimentally realized PHIL nonlinearity provides sufficient precision and monotonicity for neuromorphic inference, achieving electronic-level performance while being derived entirely from an optical thermo-optic mechanism.

Supplementary Note 8. Time stability evaluation of nonlinear response

To characterize the nonlinear response of the PHIL device and assess its resonance stability, we performed repeated modulation tests under open-loop operation, without any active feedback control. The CS power was periodically modulated while monitoring the probe transmission at three resonance wavelengths: 1557.1 nm, 1563.4 nm, and 1569.8 nm.

Supplementary Fig. 12 present the full time-resolved transmission traces. All three channels show highly repeatable thermo-optic modulation with no measurable baseline drift or phase distortion over multiple modulation cycles, confirming the intrinsic thermal stability of the device.

To further assess long-term stability, repetitive modulation was conducted for 1 hour at different wavelengths. The resulting modulation amplitude (ΔT) was analyzed statistically and plotted as mean ± σ in Supplementary Fig. 13. The small standard deviation (< ±1.7 %) corresponds to minimal wavelength drift. This long-term consistency further demonstrates the passive thermal robustness of the PHIL structure.

Supplementary Note 9. Accumulation of optical signals over wavelength with programmable transfer functions

In addition to the demonstration of linear summation of incoherent control signals and all-optical non-linear operation to the integrated time-multiplexed signals, we proceed to vary the wavelength of probe signals as discussed in the main text and successfully applied different activation functions to the incoherent summation results (Supplementary Fig. 14).

Supplementary Note 10. PHIL footprint calculation and scaling-up estimation

As shown in Supplementary Fig. 1, the PHIL architecture consists of a racetrack microring resonator (radius: 30 μm) connected via a compact 1:2 MMI splitter and waveguide S-bends. The thermo-optic phase-shifter arms used were intentionally elongated for tuning experiments and are not essential to the core PHIL function and can be replaced with compact directional bends in the final system.

We base our area estimation on the actual fabricated layouts from our experimental devices, supported by optical microscope and SEM images.

PHIL unit:

The layout footprint includes:

1. A 30 μm-radius microring: A_{ring} = ~0.003 mm^2.
2. A 1:2 MMI splitter (75−90 μm in length, 5.5−6 μm in width): A_{MMI} = ~0.0005 mm^2.
3. Routing and coupling bends occupying the remainder of the layout footprint.

Accounting for fabrication spacing rules and routing clearance, the overall footprint of one PHIL unit is estimated at:

A_{PHIL}=0.009 mm^2(conservative bounds: 0.008−0.010 mm^2).

This estimate is consistent with standard IMEC PDK and similar silicon photonics foundries. All area and throughput calculations in the main text are based on this conservative figure.

Modulator array estimation:

To ground footprint projections in realistic foundry components, we assume applying Si-Ge electro-absorption modulators (EAMs) operating at ≈ 50 Gbaud[1], each with device length 170 μm and effective width 5 μm. For both input encoding and weighting, we assume using two cascaded EAMs per wavelength channel.

The per-EAM area is 170 μm×5 μm=850 μm^2=0.00085 mm^2. Thus, with an estimated gap at 5 μm between two cascaded EAMs, the two-EAM cascade occupies around 0.001725 mm^2 before routing/contacts. Including conservative overhead for electrodes, vias, and bends, we budget 0.0022–0.0026 mm^2 per wavelength channel.

1) Per-EAM area: A_{EAM}=170 μm×5 μm=850 μm^2=0.00085 mm^2
2) Two-EAM cascade (encode + weight): A_{2EAM}=2×0.00085=0.0017 mm^2
3) Layout overhead (electrodes/contacts/bends): 0.0022–0.0026 mm^2 per channel.

With one PHIL shared across N_λ wavelengths, the tile area is $A_{PHIL}+N_\lambda A_{ch}$ with A_{PHIL}=0.009 mm^2 and A_{ch}=0.0024 mm^2 (ranges 0.008−0.0100 and 0.0022−0.0026 mm^2).

Die-area examples:

40-WDM channels: A≈40×0.0024 + 0.009 ≈0.105 mm^2

We believe these additions give the reader both (a) the concrete physical dimensions of the demonstrated device and (b) a transparent, quantitative view of how the architecture scales to a full matrix.

Supplementary Note 11. Insertion loss analysis

The insertion loss (IL) of PHIL devices depends on both wavelength and the number of integrated nanoheaters. To quantify the intrinsic loss of the reported configuration ($R = 30$ μm, 9×2 nanoheaters), devices of identical design were characterized and compared against a nanoheater-free reference fabricated with the same coupler and routing geometry.

Unlike conventional add–drop microrings, where nearly all outcoupled light is collected from a single port, the PHIL design symmetrically couples counter-propagating modes into two ports. In the present setup, only one port is collected, introducing an inherent ~3 dB coupling penalty independent of nanoheater absorption.

After accounting for this baseline, the measured IL of the reported PHIL devices is ~1.7 dB at the non-absorptive wavelength (λ_{PS}) and ~6 dB at the absorptive wavelength (λ_{CS})

(Supplementary Fig. 15a). The additional attenuation at λ_{CS} arises from localized optical absorption within the nanoheater array, while probe-wavelength losses remain below ~2 dB, indicating good transparency and minimal parasitic dissipation.

3D FDTD simulations of a compact PHIL ring ($R = 4$ μm) further confirm the strong dependence of insertion loss on the number of integrated nanoheaters (Supplementary Fig. 15b). At the absorptive wavelength (λ_{CS}), the IL increases rapidly with heater count, reaching ~11.5 dB at $N = 9 \times 2$, reflecting cumulative absorption from the expanding nanoheater array. In contrast, at the non-absorptive wavelength (λ_{PS}), IL remains low (<2 dB) and scales slowly with N, consistent with minimal modal overlap and negligible absorption. The simulated attenuation contrast therefore increases with heater number, reaching ~10 dB at $N = 9 \times 2$, in good agreement with experimental trends. The higher simulated IL compared to measurement (~11.5 dB vs. ~6 dB) arises mainly from stronger field confinement in the smaller simulated ring and fabrication-related variations that modify the heater–mode overlap.

These results confirm that the primary source of excess loss arises from localized absorption at the selected wavelength. The intrinsic nanoheater-related loss remains low (~1.7 dB at the probe wavelength) for the reported 9×2configuration, demonstrating that the PHIL architecture can achieve strong pump-selective absorption while maintaining low insertion loss, which is an essential feature for scalable, wavelength-multiplexed all-optical modulation and computing.

Supplementary Note 12. Thermal crosstalk evaluation

Finite-difference thermal simulations (Lumerical HEAT module) were performed to evaluate the spatial and temporal temperature distribution induced by optical excitation of the PHIL nanoheater array. A 150-ns optical pulse was applied to locally heat the absorptive region. The resulting transient-state temperature profile (Supplementary Fig. 16) shows a highly localized hotspot centred on the nanoheater array, with a maximum temperature rise of ~21 K above ambient. The temperature rapidly decays radially, dropping below 5 K at a distance of ~1 μm and below 1 K beyond ~2 μm from the heater centre, indicating minimal thermal crosstalk to adjacent photonic components. This localized heating corresponds to an estimated optical phase shift of ~0.025 π for the reported device configuration, consistent with experimental modulation levels.

Comparison with Conventional Thermo-Optic Phase Shifters:

Unlike standard thermo-optic phase shifters which typically rely on extended metallic heaters[2,3] (e.g., several tens of microns) and additional driving electronics that introduce parasitic thermal load, our nanoheater design is highly localized (sub-micron scale) and optically actuated, eliminating the need for electrical contacts. This will significantly reduce the total thermal budget and the spatial extent of heat diffusion. We further note that the standing-wave-enhanced spatial

confinement of optical heating in our system intrinsically favours localized thermal interaction with minimal lateral diffusion. Combined with established foundry-compatible strategies for suppressing thermal crosstalk, such as undercutting and thermal isolation trenches, this design supports scalable deployment in densely integrated photonic arrays.

Supplementary Note 13. Energy efficiency

Energy estimation:

At 50 GHz repetition rate,

$$T_{\mathrm{pulse}} = \frac{1}{50}\mathrm{GHz} = 20\ \mathrm{ps}.$$

Within a 20 ns integration window:

$$N_{\mathrm{pulses}} = 20\ \mathrm{ns}/\ 20\ \mathrm{ps} = 1000\ \mathrm{pulses}.$$

Assuming half correspond to logical "1" and half to logical "0", approximately 500 active pulses contribute to the energy accumulation.

The nonlinear activation is triggered at an optical power of ≤ 2.6 mW.

Energy per pulse:

$$E_{\mathrm{CS,pulse}} = 52\ \mathrm{fJ}.$$

Hence, each "1" pulse costs ~52 fJ.

Total control-signal energy per 20 ns window:

$$E_{\mathrm{CS,total}} = 500 \times 52\ \mathrm{fJ} = 26000\ \mathrm{fJ} = 26\ \mathrm{pJ}\,.$$

Probe-signal contribution (0.1 mW steady across the window):

$$E_{\mathrm{probe}} = 2\ \mathrm{pJ}.$$

Therefore, the total optical energy per integration window is:

$$E_{\mathrm{total}} = E_{\mathrm{CS,total}} + E_{\mathrm{probe}} = 28\ \mathrm{pJ}.$$

Normalized Energy per MAC:

Each 20 ns integration window accumulates approximately 500 operations (MAC equivalents).

$$E_{\mathrm{per-operation}} = 28\mathrm{pJ}/500 = 0.056\mathrm{pJ} = 56\ \mathrm{fJ}$$

The PHIL system uniquely performs temporal integration entirely in the optical domain while applying programmable nonlinearity, which is a functionality not achievable by other all-optical technologies. Its opto-thermal design allows summation of thousands of ultrafast optical inputs over a ~130 ns thermal window, making it inherently suitable for neuromorphic computing tasks requiring accumulation and thresholding over time.

Compared to phase-change materials (PCM), PHIL is volatile but enables real-time, analogue tunability. PCMs are non-volatile and ideal for static reconfiguration, yet their

nanosecond-scale switching hinders high-speed integration. Relative to ultrafast Kerr or 2D material devices, PHIL trades off switching speed for the ability to perform analogue accumulation and nonlinear activation in a single passive platform, leveraging its slow-heat-based integration dynamics.

In summary, PHIL occupies a distinct niche among all-optical integrative computing schemes: it offers repeatable, energy-efficient accumulation with multi-bit analogue precision, balancing performance and tunability where other mechanisms prioritize either speed or non-volatility. While its thermal time constant (~130 ns) is slower than sub-picosecond nonlinearities, integration occurs over this window independent of the incoming pulse rate, enabling compatibility with ultrafast (50 GHz) pulse streams and WDM operation, a regime difficult to access using carrier-dynamics-based photonic neuromorphic hardware.

Supplementary Figures

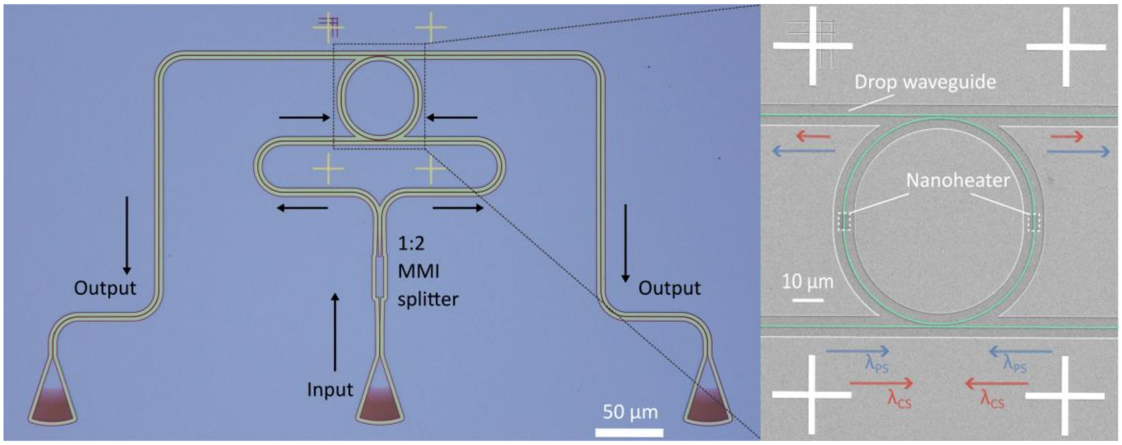

Supplementary Fig. 1 Optical microscope image and SEM image of PHIL-loaded ring resonator. The input light is split by a 1:2 MMI splitter into two counter-propagating paths that excite the microring resonator. The clockwise and counterclockwise waves interfere to form a standing-wave field, with nanoheaters positioned at the antinodes for localized optical absorption. The out-coupled signals are symmetrically distributed between the two output ports; only one port is collected in the present setup, resulting in an intrinsic ~3 dB coupling penalty independent of absorption losses.

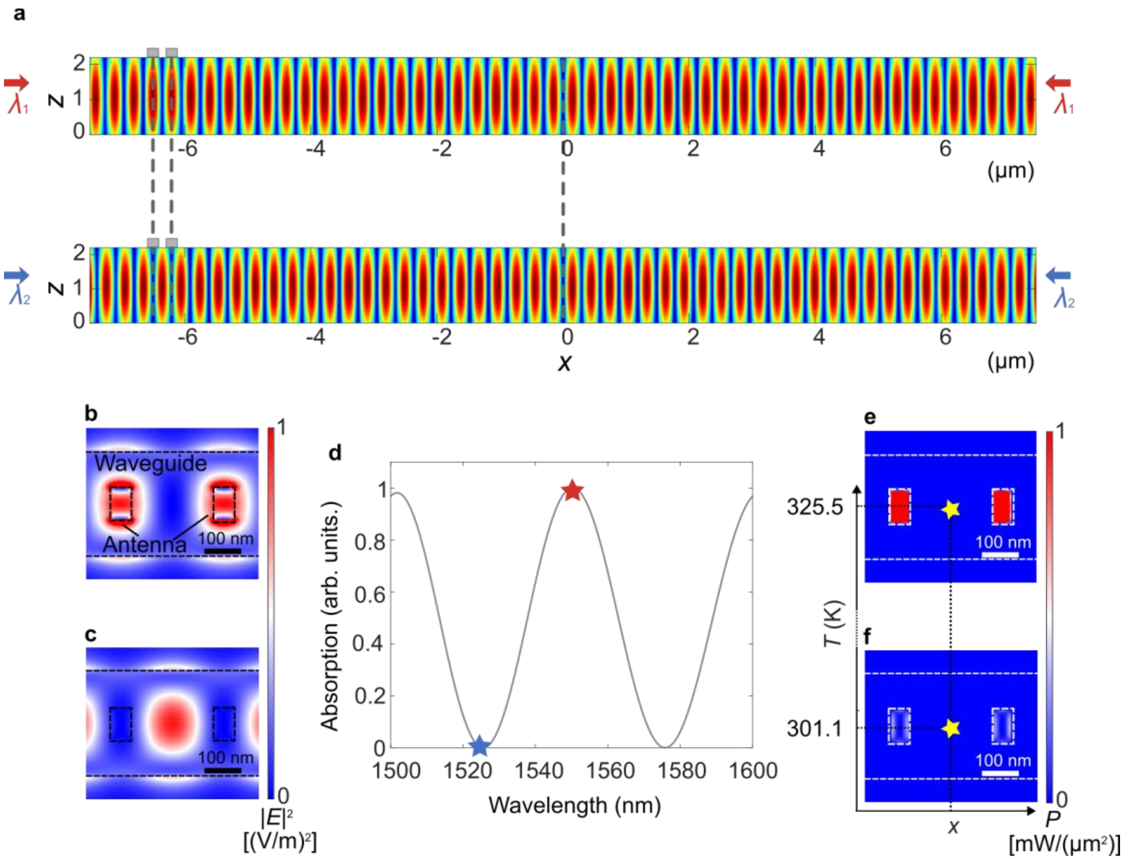

Supplementary Fig. 2 Across-wavelength photon interactions via the thermo-optic effect. a Input light with equal intensities is sent into silicon waveguide from opposite directions. Two absorptive nano-antennas are designed to spatially match the anti-nodes of light λ_1 (1550.3 nm) while overlapping with the nodes of light λ_2 (1525.6 nm). **b, c** Normalized E-field of different wavelengths at the antennas. Titanium antennas of 20 nm thickness, 50 nm width and 250 nm length are placed on a rib waveguide with 500 nm width in the simulation. **b** Two absorptive nano-antennas are designed to spatially match the anti-nodes of λ_1, **c** while overlapping with the nodes of λ_2. **d** Here the total absorption experienced by λ_1 is 17 times higher than that of $\lambda_2$where the structure is nearly transparent. **e, f** Power absorbed per unit area by antennas with 1-mW input power. Inset Fig. indicates that heat generated by the absorption of light in the antenna region raises the temperature (K) of the surrounding waveguide, generated with *Lumerical* HEAT.

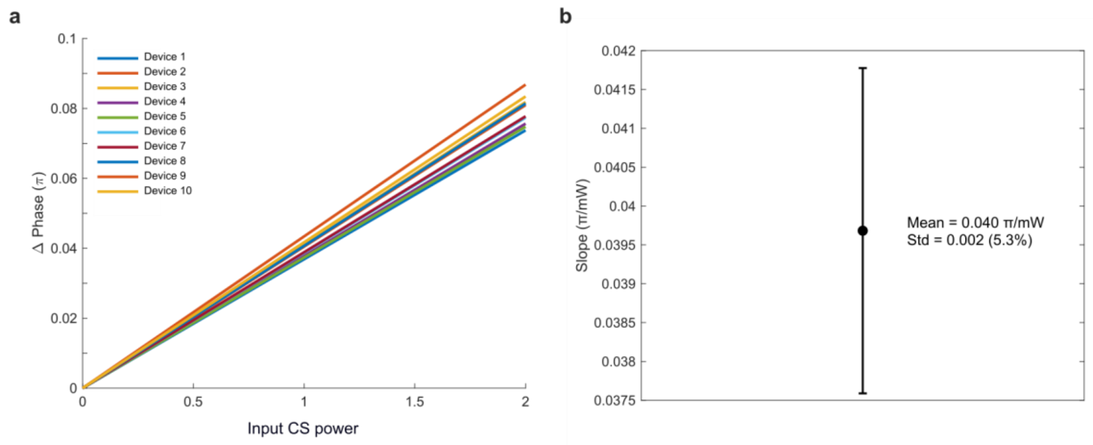

Supplementary Fig. 3 Uniformity of optical phase tuning across multiple microring devices. a Phase tuning response (Δ Phase vs input CS power) for ten PHIL units, each exhibiting a nearly linear thermo-optic response. **b** Statistical summary of the tuning efficiency extracted from **a**, showing the mean slope and its standard deviation across all devices (0.040 π/mW ± 0.002 π, corresponding to 5.3% variation). The small spread confirms highly uniform phase-tuning behaviour within the fabricated array.

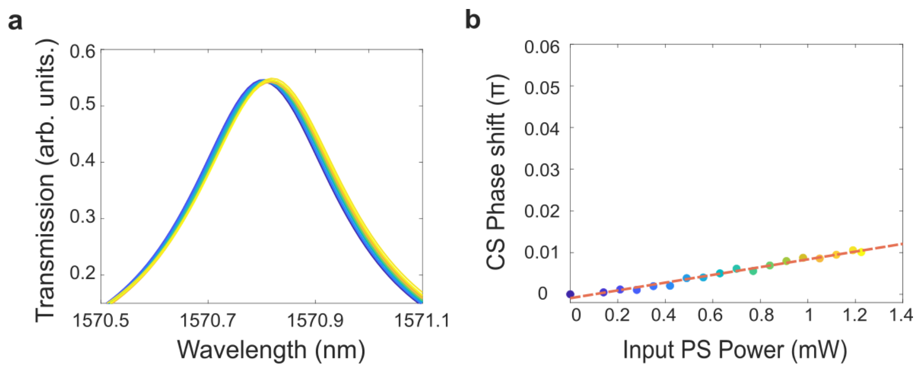

Supplementary Fig. 4 Spectral response characterization with modulating probe signal. a Spectral response collected at odd mode modulated by even mode. **b** Pump at even mode has a weak effect at 0.0087 π/mW.

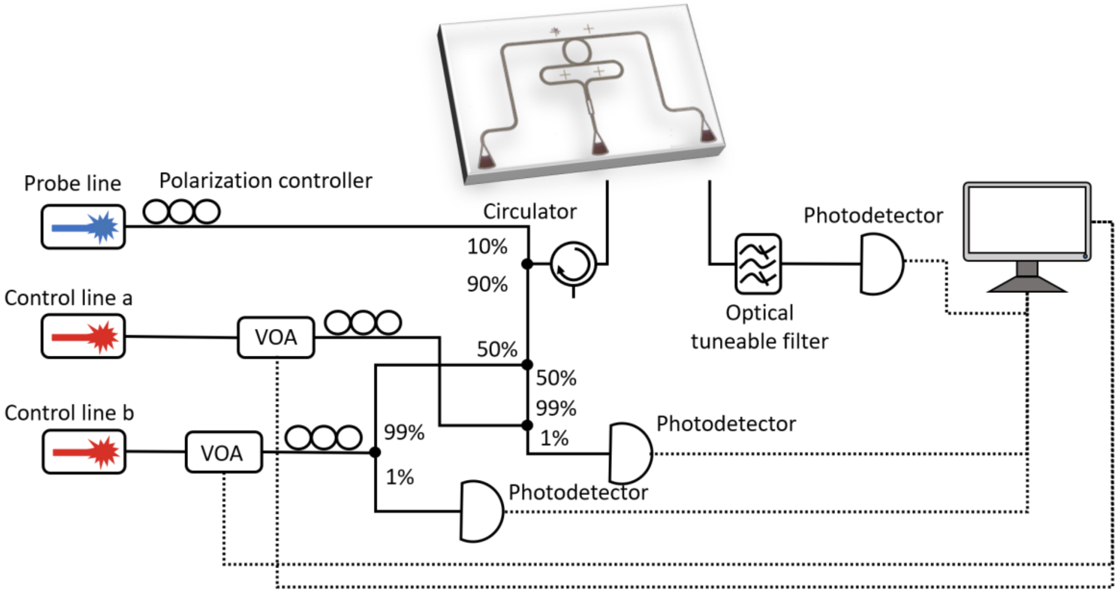

Supplementary Fig. 5 Experiment set-up for accumulation operations across wavelengths.

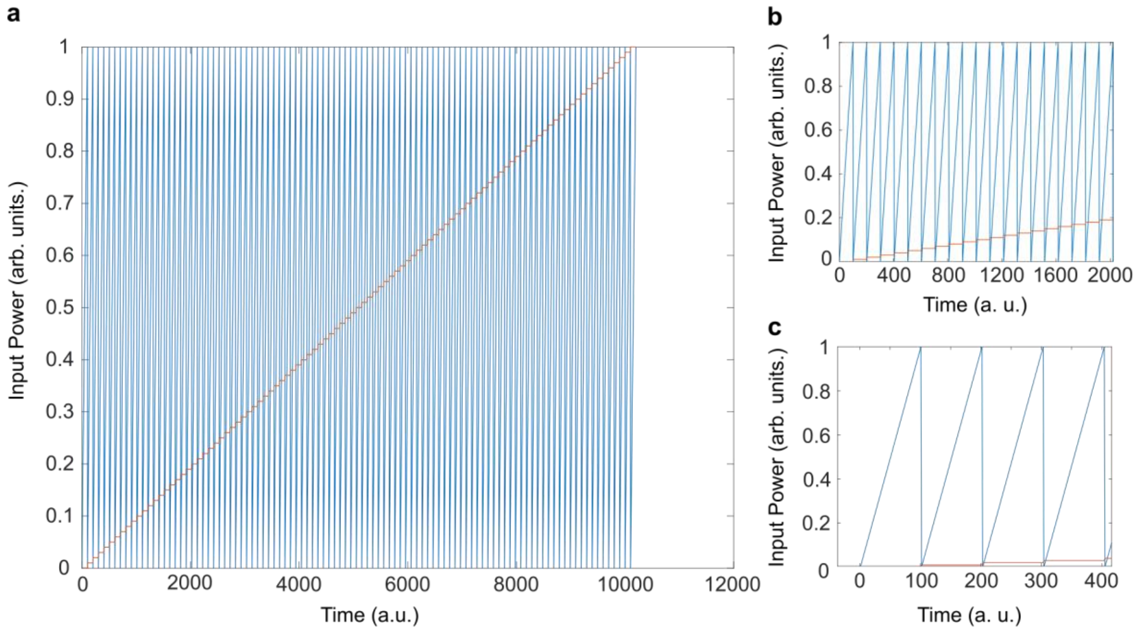

Supplementary Fig. 6 Input power of control signals through the measurement. **a** Each control signal is assigned 101 power steps, control signal a (red line) moves one power step at every 101 time-steps while control signal b (blue line) will move one power steps at each time step. 10,201 addition events were measured through the experiment. **b, c** Zoomed-in view at selected time steps.

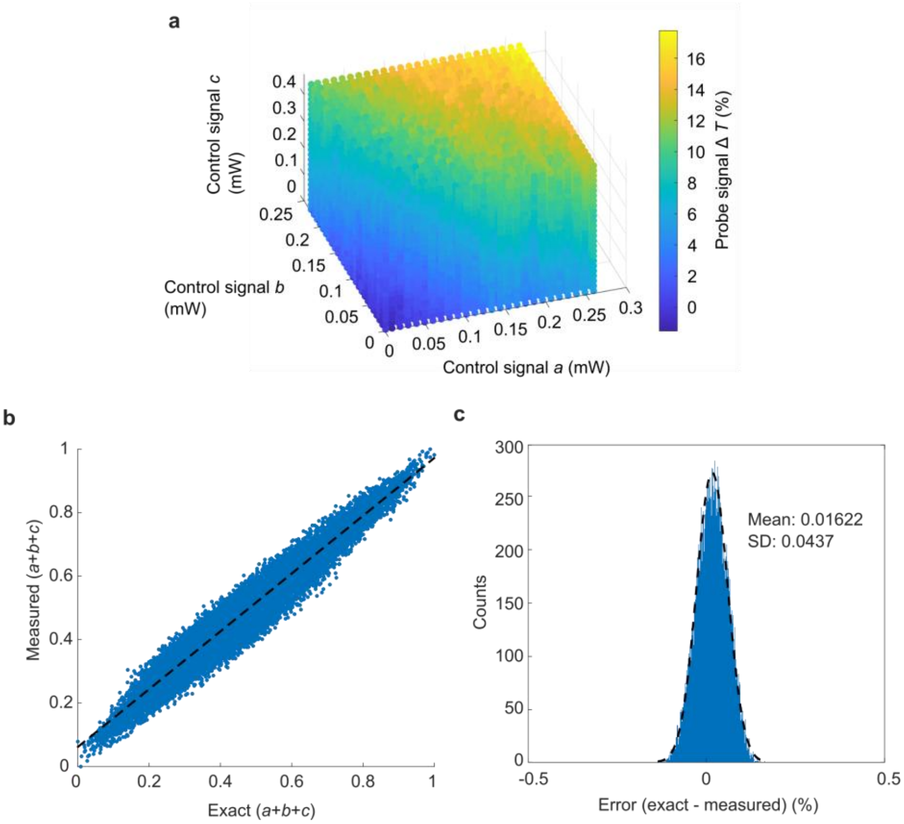

Supplementary Fig. 7 All-optical summation across 3 wavelengths. **a** Measured nonlinear summation surface of the probe transmission (ΔT) as a function of three independent control-signal (CS) powers (a, b, c) at distinct. The probe output increases monotonically with total optical power, confirming additive thermal accumulation across multiple wavelengths. **b** Comparison between the measured output and the ideal additive response, showing excellent linear correlation. **c** Histogram of the deviation between measured and expected responses, yielding a standard deviation of σ = 0.0437 (mean = 0.0162), corresponding to an effective precision of approximately 4.5 bits. These results demonstrate that the PHIL architecture maintains high linearity and low crosstalk even under simultaneous multi-wavelength excitation.

299

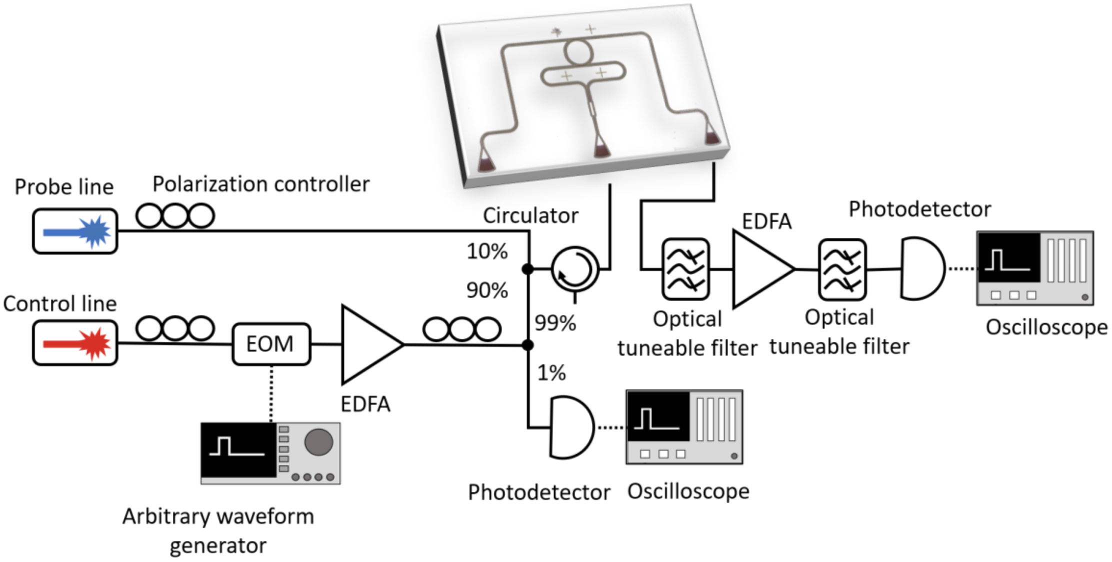

Supplementary Fig. 8 Experiment set-up for time-scale response characterization.

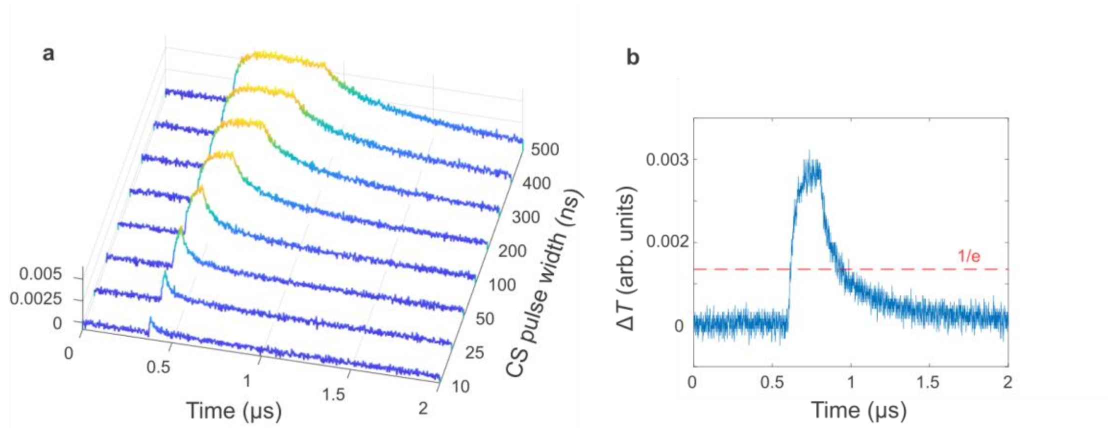

Supplementary Fig. 9 Square pulse response characterization. a Transmission change (Δ *T*) at probe signal in response to input control signal with pulse width from 10 ns to 500 ns. **b** Leaky time constant τ (130 ns) is calculated as the time for output to decay to 1/e of its initial value when the input is removed. (Input CS pulse width: 200 ns)

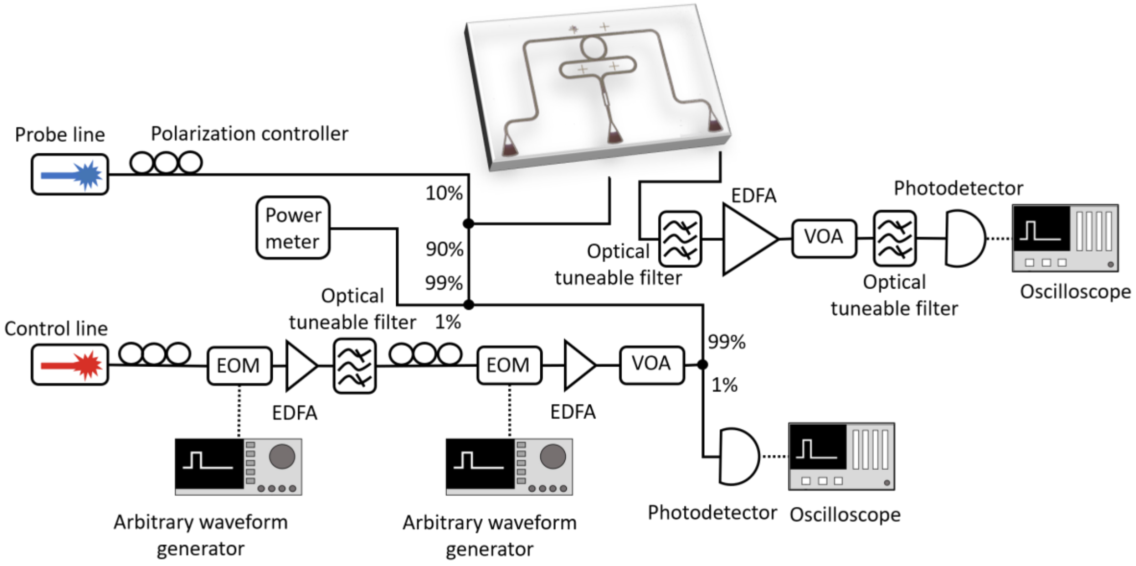

Supplementary Fig. 10 Experiment set-up for integrating time-resolved signal.

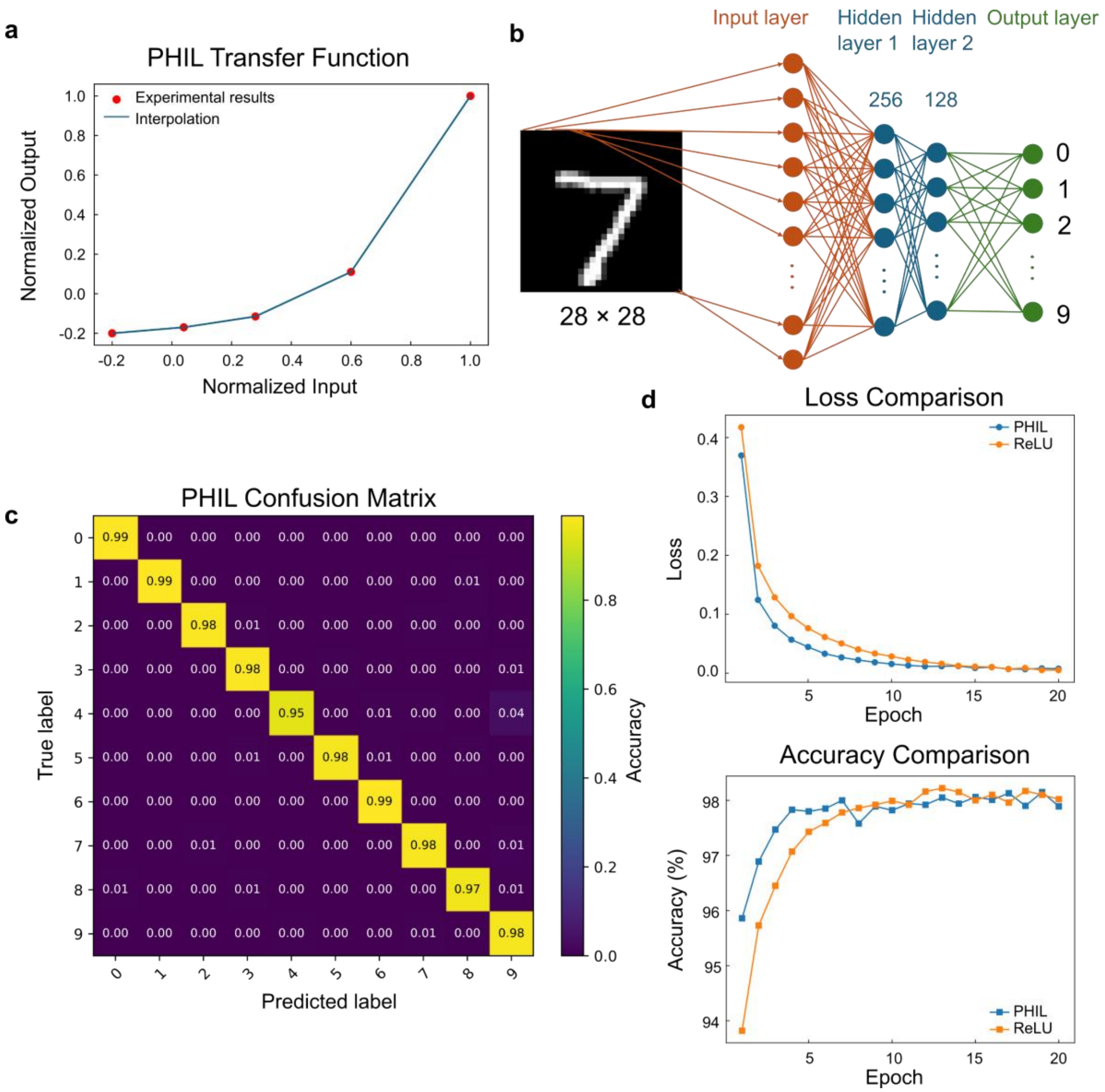

Supplementary Fig. 11 Simulation of PHIL activation function in a neural network for handwritten digit recognition. **a** Experimentally measured PHIL transfer curve (red circles) and interpolated nonlinear activation function (blue line) used for the network simulation. **b** Schematic of the fully connected neural network architecture (784–256–128–10) used for MNIST handwritten digit classification, where the experimental PHIL activation replaces the standard electronic ReLU function. **c** Confusion matrix of the PHIL-activated network showing accurate digit recognition across all classes with classification accuracy of 97.2 %, compared to 97.8 % for the ReLU baseline. **d** Training loss and validation accuracy versus epoch for PHIL and ReLU activations, demonstrating nearly identical convergence behaviour.

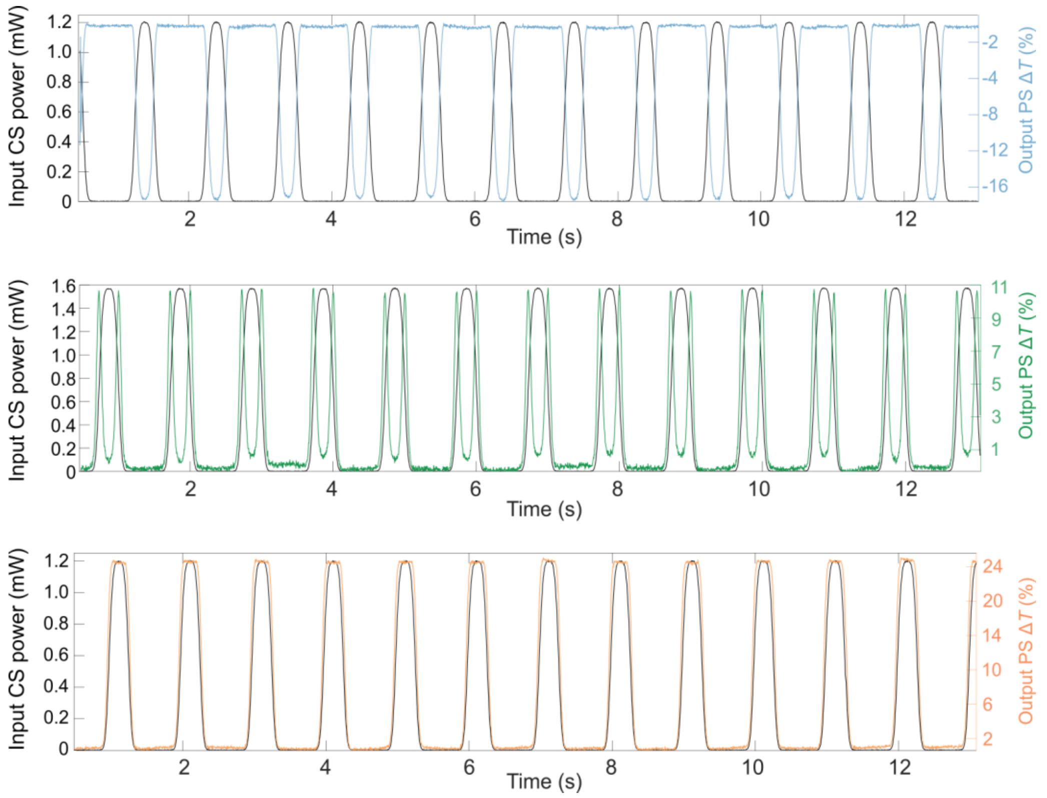

Supplementary Fig. 12 Non-linearity measurements over multiple cycles.

305

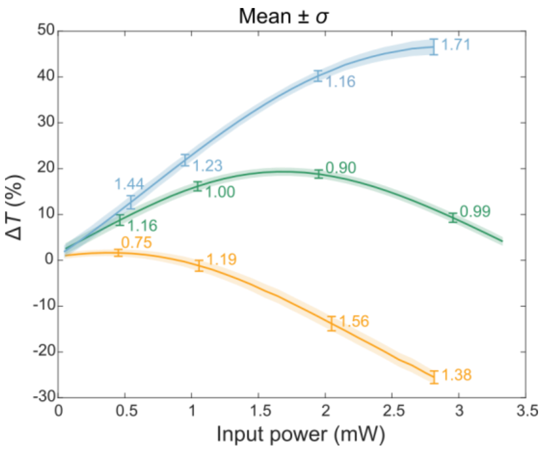

Supplementary Fig. 13 Long-term (1h) stability of the PHIL device under repeated CS modulation. Statistical analysis of the modulation amplitude (ΔT) as a function of input CS power for the three wavelengths, plotted as mean ± σ over one-hour continuous operation. The minimal deviation (< ±1.7 %) excellent thermal and spectral stability of the device in open-loop conditions.

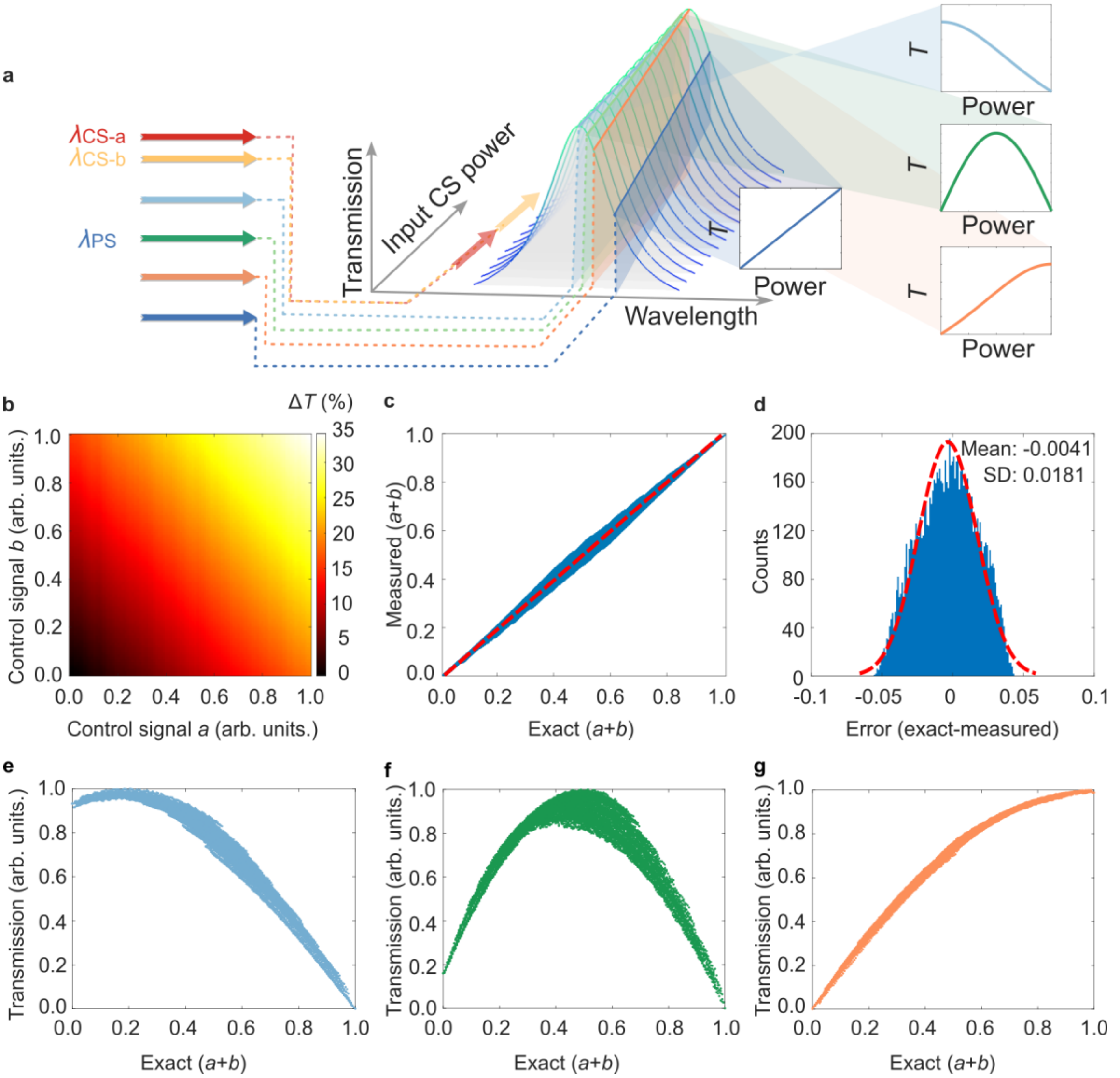

Supplementary Fig. 14 Across-wavelength accumulation with optically programmable transfer functions. a Concept of the across-wavelength accumulation, where optical power of incoherent control signals is accumulated in heat, shifts the spectrum by thermo-optic effect, and encode onto the amplitude of a probe wavelength by selectively applying transfer function with exploring the spectrum shape of MRR. **b** Linear addition of signals carried by the intensity of two incoherent CS to the PS, and addition results are encoded within $\Delta T = (T - T_0)/ T_0$, which is the change in transmission of the level T with respect to the baseline T_0. **c** Measured addition results (calculated from normalized transmission variation in PS) compared with the expected condition. **d** Error of the addition operation calculated from subtracting the measured addition results from the originally programmed addition results from 10210 addition events. **e, f, g** Non-linear functions applied on the across-wavelength summation results, obtained from different probe wavelengths in a single all-optical unit

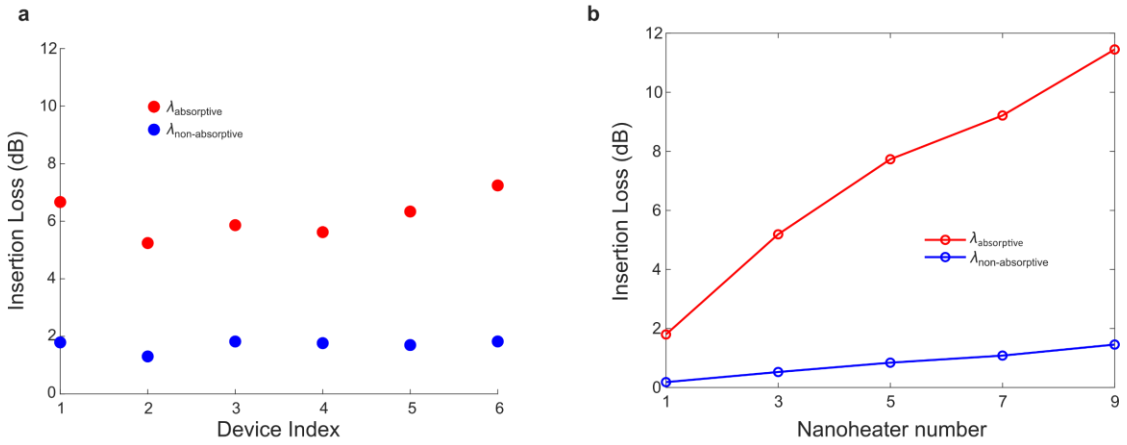

Supplementary Fig. 15 Measured and simulated insertion loss of the PHIL architecture. a Measured insertion loss of the reported PHIL devices (9×2 nanoheaters), referenced to identical designs without integrated nanoheaters. The loss at the absorptive wavelength ($\lambda_{\text{absorptive}}$) is significantly higher than that at the non-absorptive wavelength ($\lambda_{\text{non-absorptive}}$), confirming localized optical absorption by the embedded heaters. **b** Simulated insertion loss in a compact PHIL configuration as a function of the number of nanoheaters (1×2, 3×2, 5×2, 7×2 and 9×2). Insertion loss increases rapidly with heater count at the absorptive wavelength, reaching ~11.5 dB at $N = 9 \times 2$, due to cumulative

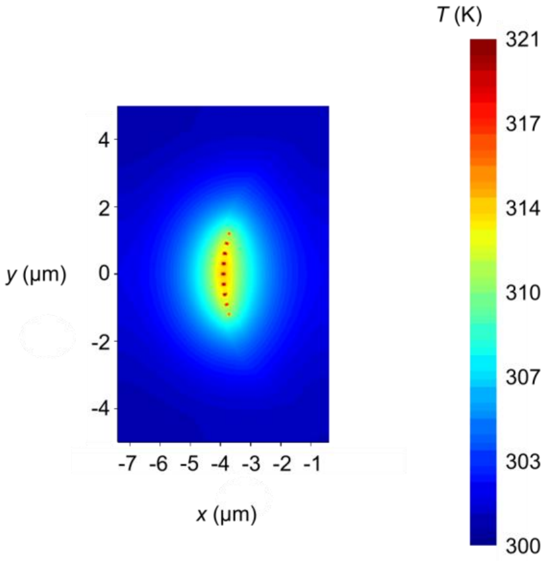

Supplementary Fig. 16 Simulated temperature profile and thermal crosstalk evaluation of PHIL nanoheaters. Transient-state temperature distribution obtained from finite-difference heat simulations (Lumerical HEAT module) under a 150-ns optical pulse excitation at the PHIL region. The peak temperature rise is confined within ~2 μm around the nanoheater array, indicating minimal thermal crosstalk to adjacent photonic components. Beyond 2 μm separation, the temperature rise falls below 1 K, confirming good thermal isolation suitable for large-scale photonic integration.

Supplementary Tables

Supplementary Table 1 |Comparison of All-Optical Light-Controlled Technologies

Technology	Energy per Operation	Speed (Response/BW)	Precision	Optical Temporal Integration + Nonlinear Activation	Ref
**Photonic Heater-in-Lightpath (PHIL) Integrator**	~56 fJ	~130 ns thermal time constant (≈ 1.2 MHz bandwidth). Can integrate ultrafast (~50 GHz) optical pulses over this window	~5-bit analog resolution demonstrated	Yes	**This work**
**Phase-Change Material (PCM)**	~tens of pJ	~10–100 ns optical pulse required for phase change	Multi-level analog phase possible (~5-bit)[4]	No	Li *et al.*, 2019[4]; Farmakidis *et al.*, 2019[5]; He *et al.*, 2024[6]
**Free-Carrier Optical Injection**	~hundreds of fJ	~hundreds of ps (carrier lifetime limited)	*	No	Preston *et al.*, 2008[7]; Shi *et al.*, 2022[8]
**2D Material All-Optical Modulator**	~tens of fJ	~hundreds of fs	*	No	Ono *et al.*, 2020[9]
**Semiconductor optical amplifier (SOA)**	~3 pJ (~100 pJ, if electrical bias included)	~hundreds of ps	Sigmoid activation fitted to logistic function with high agreement (NRMSE < 0.08, dynamic range ~27 dB for cross-connect networks)	Yes-but with electrical bias	Mourgias-Alexandris *et al.*, 2019[10]; Kravtsov *et al.*, 2011[11]; Shi *et al.*, 2020[12]

* Not reported

Supplementary References

1. Giamougiannis, G. *et al.* Neuromorphic silicon photonics with 50 GHz tiled matrix multiplication for deep-learning applications. *AP* **5**, 016004 (2023).
2. Harris, N. C. *et al.* Efficient, compact and low loss thermo-optic phase shifter in silicon. *Opt. Express, OE* **22**, 10487–10493 (2014).
3. Jacques, M. *et al.* Optimization of thermo-optic phase-shifter design and mitigation of thermal crosstalk on the SOI platform. *Opt. Express, OE* **27**, 10456–10471 (2019).
4. Li, X. *et al.* Fast and reliable storage using a 5 bit, nonvolatile photonic memory cell. *Optica, OPTICA* **6**, 1–6 (2019).
5. Farmakidis, N. *et al.* Plasmonic nanogap enhanced phase-change devices with dual electrical-optical functionality. *Science Advances* **5**, eaaw2687 (2019).
6. He, Y. *et al.* Energy-Efficient Integrated Electro-Optic Memristors. *Nano Lett.* **24**, 16325–16332 (2024).
7. Preston, K., Dong, P., Schmidt, B. & Lipson, M. High-speed all-optical modulation using polycrystalline silicon microring resonators. *Applied Physics Letters* **92**, 151104 (2008).
8. Shi, Y. *et al.* Nonlinear germanium-silicon photodiode for activation and monitoring in photonic neuromorphic networks. *Nat Commun* **13**, 6048 (2022).

9. Ono, M. *et al.* Ultrafast and energy-efficient all-optical switching with graphene-loaded deep-subwavelength plasmonic waveguides. *Nat. Photonics* **14**, 37–43 (2020).
10. Mourgias-Alexandris, G. *et al.* An all-optical neuron with sigmoid activation function. *Opt. Express, OE* **27**, 9620–9630 (2019).
11. Kravtsov, K., Fok, M. P., Prucnal, P. & Rosenbluth, D. Ultrafast All-Optical Implementation of a Leaky Integrate-and-Fire Neuron. *Optics express* **19**, 2133–47 (2011).
12. Shi, B., Calabretta, N. & Stabile, R. Deep Neural Network Through an InP SOA-Based Photonic Integrated Cross-Connect. *IEEE Journal of Selected Topics in Quantum Electronics* **26**, 1–11 (2020).
